# Development of the Electric Equivalent Model for the Cytoplasmic Microinjection of Small Adherent Cells

**DOI:** 10.3390/mi8070216

**Published:** 2017-07-08

**Authors:** Florence Hiu Ling Chan, Runhuai Yang, King Wai Chiu Lai

**Affiliations:** 1 Department of Mechanical and Biomedical Engineering, City University of Hong Kong, Hong Kong, China; florencehlchan@gmail.com; 2 Department of Biomedical Engineering, Anhui Medical University, Hefei 230032, China; yrunhuai@foxmail.com; 3 Centre for Robotics and Automation, City University of Hong Kong, Hong Kong, China

**Keywords:** cell cytoplasmic microinjection, small adherent cells, equivalent-circuit model

## Abstract

A novel approach utilizing current feedback for the cytoplasmic microinjection of biological cells is proposed. In order to realize the cytoplasmic microinjection on small adherent cells (diameter < 30 μm and thickness < 10 μm), an electrical model is built and analyzed according to the electrochemical properties of target cells. In this study, we have verified the effectiveness of the current measurement for monitoring the injection process and the study of ion channel activities for verifying the cell viability of the cells after the microinjection.

## 1. Introduction

Cell injection is a promising micromanipulation method in the biological field [1]. Efficient tools for precisely injecting drugs into cells are of significant value to the development of novel therapeutics [2], and are also important for the study of cellular activity in biological fields [3]. Progresses in biological engineering such as drug screening and transfection require the precise manipulation of single biological cells [4]. Several methods, including drug delivery by either nano-vehicles or nanoparticles, may be used to penetrate the cell membrane and perform intracellular delivery [5,6]. In addition, viral vector-based methods are very effective for injecting the target nuclide acid into various kinds of cells, ranging from the ex vivo transduction of hematopoietic cells to in vivo transgene expression through the optimization of tissue-specific cells. However, safety remains a great concern for gene therapies using the viral vector [7]. Besides, a nano-needle is very efficient for delivering different molecules and materials including antibodies, quantum dots, and nanoparticles to primary hippocalmpus neuron cells and NIH3T3 fibroblast cells, which both have a diameter of around 100 μm [8]. Unfortunately, the injection performance on small adherent cells using the nano-needles is unknown at present.

A micro-pipette-based microinjection is a process for the delivery of target materials by penetrating living cells using a micropipette. It has been proven to work effectively and has the advantages of a precise control of the delivery dosage and a high transduction efficiency [9]. However, the drawback of pipette-based methods is the need for a well-trained operator, thus requiring a lot of time and money [10]. This led to the demand of an automatic pipette-based cell microinjection in order to increase the success rate and reduce human errors [11]. Several automated cell injection approaches with different kinds of feedbacks were reported, including force feedback, impedance feedback, and visual feedback. The microNewton force feedback approach was reported to largely improve the work efficiency and success rate of microRNA and DNA injections to suspended mouse embryos (around 100 μm in diameter) and zebrafish cells (1.3 mm in diameter) [12]. For smaller cells, the penetration force analysis of several cell lines, which are L929, HeLa, 4T1, and TA3 HA II cells, was demonstrated using atomic force microscopy (AFM) [2], which helps the researcher to understand the cell mechanics. Impedance feedback for a robot-assisted cell microinjection system performed on zebrafish fish embryos was introduced to enhance the accuracy of the system feedback [2,13]. A vision-based force measurement for cell microinjection was performed on mouse oocytes (around 100 µm in diameter) and drosophila embryos (around 500 μm in diameter) [14]. A visual-based system with impedance control for cell injection was reported to perform successfully on Zebrafish embryos [15]. Some researchers suggested that the visual feedback may be a possible solution for the micro-injection of small cells with a diameter of less than 100 µm [16]. However, another challenge which arises from visual feedback is the difficulty in observing the contact between the tip and the cell to determine the penetration status. In recent years, more genetic research has driven the demand of an automated cell microinjection system, and some in vitro cell microinjection systems were reported in a review [17]. All of the microinjection systems mentioned are for suspending cells in an oval shape. Nevertheless, some typical human cells are adherent and small, such as neuroblastoma cells and human epithelia cells. Recently, an automated injection system for suspending small cells (diameter < 30 µm) was reported, which was operated with a microfluidic cell holder chip [11,18]. Small adherent cells are of importance in cell biology because of the size of human cells, which usually ranges from 7 µm to 25 µm, and also cell transfection using human cells with the precise delivery of target materials is challenging to operate, but there is great demand for it to be automated [11]. In the past, there have been quite a number of researches about the automated injection of large and round cells such as the zebra fish embryo [1,4,9,19,20]. In contrast, there are only a few number of researches talking about the automated injection of small adherent cells [21]. For instance, SHSY-5Y cells (human neuroblastoma cells) are used frequently for studying Alzheimer’s disease. HEK-293 cells (human embryonic kidney cells) are used frequently for studying gene therapy. However, it is more difficult to implement either the manual or automated injection on human cells due to their small size.

To overcome the challenges, a current feedback approach is proposed to work for automating the micro-injection of the neuronal cell line (<30 μm). In this study, an equivalent electrical model was established to monitor two situations. The real time current response during the microinjection was studied for the effectiveness of the injection. In addition, the current trace under voltage stimulus was recorded in order to verify the viability of the cells after microinjection.

## 2. The Electric Equivalent Model for Cytoplasmic Microinjection and Cell Viability Verification

**Figure 1:**
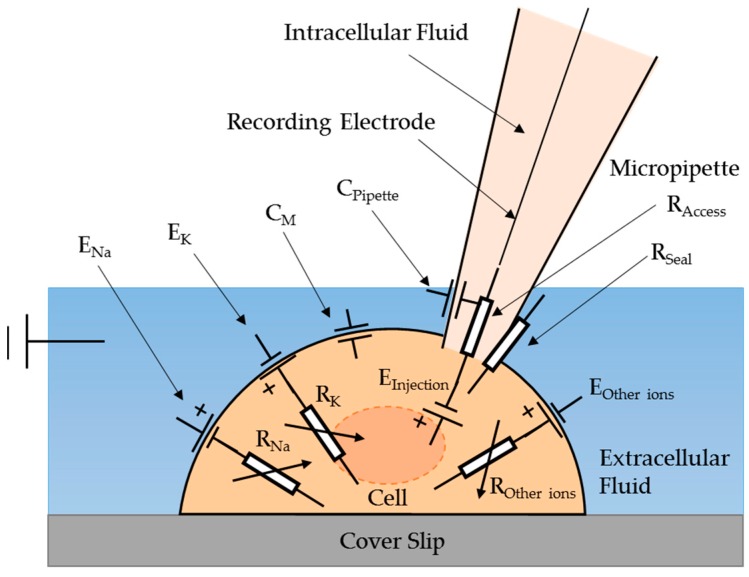
The electric equivalent model of the cell ctoplasmic injection.

In order to monitor the electrical signal of biological cells for microinjection, an electric equivalent model of a cell cytoplasmic microinjection with a micro-pipette has been proposed, as illustrated in Figure 1. A cell interacting with a micro-pipette can be regarded as an electrical circuit containing several electrical components. *E_Na_*, *E_K_*, and *E_Other ions_* are the equilibrium (Nernst) potentials of Na^+^, K^+^, and other ions between the cell membrane. In normal condition, *E_K_* and *E_Na_* are about −90 mV and +60 mV [22] due to the concentration difference between the outer and inner fluid of living cells. Naturally, the external fluid usually contains 0.44 M of Na+, and the internal fluid, which is the cytoplasm, usually contains 0.5 M of K^+^ and a number of unspecified anions such as phosphates, amino acids, and negatively charged proteins [23]. In this study, cells were bathed in the buffer solution as the outer fluid of living cells, called extracellular solution. Meanwhile, another solution was prepared as the inner fluid of living cells, called intracellular solution.

The capacitance (*C_M_*) of the cell membrane exists because of the different concentration of total ions between the outer and inner fluid. The glass layer structure of the pipette acts as a capacitor, *C_Pipette_*, which gives the capacitance transient curve when the micropipette is immersed in the extracellular solution. The capacitance transient curve can be neutralized by injecting the opposite capacitance current via adjustment with observation of the electrical signal. *R_Na_*, *R_K_*, and *R_Other ions_* are the variable resistances of the ionic currents contributed by Na^+^, K^+^, and other ions, respectively. Their resistances vary because the various ions (Na^+^, K^+^, and other ions) pass through the cell members when the corresponding ion channels are activated under a different stimulus [24]. *R_Seal_* is a resistance due to the formation of a tight and strong seal between the cell surface and the pipette before the microinjection. Access resistance (*R_Access_*) results from the geometry of the pipette plus any obstruction at the tip by the adherent membrane [19].

In the model, *E_Injection_* is hypothesized to exist when the intracellular solution is injected into a cell using a micropipette. It is believed that the injection will cause a significant change of the membrane potential because of the change of ion concentrations across the membrane. During the injection, the injected intracellular solution immediately mixes with the cell cytoplasm leading to a change of ion concentration in the cytoplasm, while the concentration of the extracellular solution remains unchanged. *E_Injection_* can be expressed as shown in Equation (1). *E_Cell Membrane_* can be calculated by the Nernst Equation, as shown in Equation (2):

(1) $$E_{Injection}=New\;E_{Cell\;Membrane}-\;Old\;E_{Cell\;Membrane}$$

where *New E_Cell Membrane_* is the membrane potential after the injection, and *Old E_Cell Membrance_* is the membrane potential before the injection:

(2) $$E_{Cell\;Membrane}=\overset{n}{\underset{j=0}{\sum }}\frac{RT}{ZF}ln\frac{{\left[ion_{j}\right]}_{o}}{{\left[ion_{j}\right]}_{i}}$$

where *R* is the ideal gas constant, *R* = 8.314472 J K^−1^·mol^−1^, *T* is the temperature in kelvins, *F* is Faraday’s constant (coulombs per mole), *F* = 9.648533 × 10^4^ C·mol^−1^, *[ion]_o_* is the extracellular concentration of that particular ion (in moles per cubic meter), *[ion]_i_* is the intracellular concentration of that particular ion (in moles per cubic meter), and *Z* is the number of moles of electrons transferred in the cell reaction or half-reaction.

By taking into consideration all of the ions of the intracellular solution, as mentioned in Section 3, *E_Injection_* can be expressed as shown in Equation (3).

(3) $$\begin{array}{cc} E_{cell\;membrane}= & \frac{RT}{\left(+1\right)F}ln\frac{{\left[Na^{+}\right]}_{o}}{{\left[Na^{+}\right]}_{i}}+\;\frac{RT}{\left(+1\right)F}ln\frac{{\left[K^{+}\right]}_{o}}{{\left[K^{+}\right]}_{i}}+\;\frac{RT}{\left(+2\right)F}ln\frac{{\left[Mg^{2+}\right]}_{o}}{{\left[Mg^{2+}\right]}_{i}}+\;\frac{RT}{\left(-2\right)F}ln\frac{{\left[Cl^{2-}\right]}_{o}}{{\left[Cl^{2-}\right]}_{i}}\\ & +\;\frac{RT}{\left(Z\right)F}ln\frac{{\left[Other\;ions\right]}_{o}}{{\left[Other\;ions\right]}_{i}} \end{array}$$

where $\frac{RT}{\left(Z\right)F}ln\frac{{\left[Other\;ions\right]}_{o}}{{\left[Other\;ions\right]}_{i}}\;≅0$ , since the concentration of ion is very small.

Since the major injected ions are K^+^ and Na^+^, the equation of cell membrane potential can be simplified in Equation (4).

(4) $$E_{cell\;membrane}=\frac{RT}{\left(+1\right)F}ln\frac{{\left[Na^{+}\right]}_{o}}{{\left[Na^{+}\right]}_{i}}+\;\frac{RT}{\left(+1\right)F}ln\frac{{\left[K^{+}\right]}_{o}}{{\left[K^{+}\right]}_{i}}$$

To study and verify this electrical model, the patch clamp technique was used with a low noise operation amplifier that can apply voltage stimulus for controlling the voltage potential of the cell membrane, called the membrane potential (*V_m_*). It can also acquire the total current response of the cell membrane, called the membrane current (*I_m_*).

**Figure 2:**
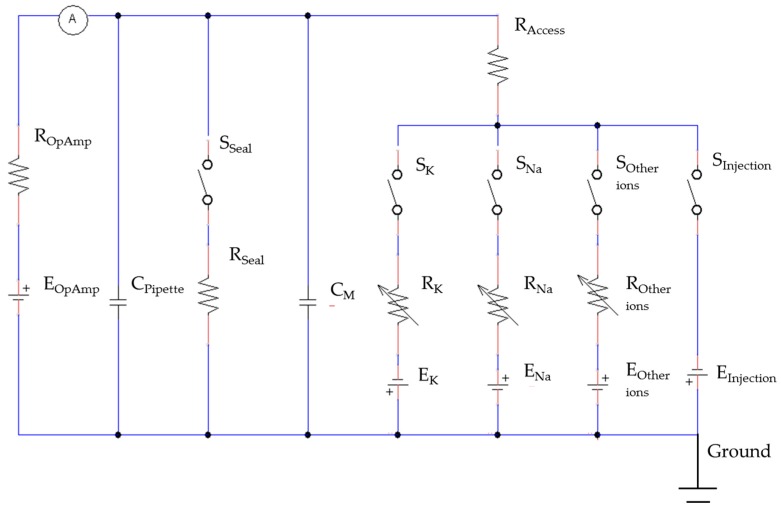
The equivalent circuit of the current feedback model for the cell cytoplasmic microinjection and cell viability verification.

When a cell is connected to the operational amplifier, the electric equivalent circuit of the cell cytoplasmic microinjection is constructed as shown in Figure 2. The operation amplifier acts as a voltage source (*E_OpAmp_*) in series with a resistor (*R_OpAmp_*) in order to apply the voltage stimulus which can evolve the ion flow along the ion channels. There are five switches in the circuit, including the switch (*S_seal_*) for the sealing resistance and the switch (*S_Injection_*) for the microinjection of the cell. In this circuit, *S_seal_* is always open as *R_Seal_* is extremely large compared with other resistances, and hence the connection to *R_Seal_* will act as an open circuit. The function of the switch (*S_Injection_*) for the microinjection of the cell is to connect the *E_Injection_* when the intracellular solution is injected into the cell. *S_K_*, *S_Na_*, and *S_Other ions_* act like the switches to connect *R_Na_, R_K_*, and *R_Other ions_*, which will be the maximum when there is the least ion channel activity, and vice versa.

In the measurement, the membrane current (*I_M_*) can be recorded. According to the circuit model, *I_M_* is composed of several components, as shown in Equation (5), including the current caused by the pipette capacitance (*I_Pipette_*), the current passing through the sealing resistance (*I_Seal_*), the current passing through the access resistor (*I_Access_*), and the current caused by the membrane capacitance (*I_c_*). *I_C_* is produced by the charge accumulation at the outer and inner membrane surface when *V_m_* changes.

(5) $$I_{m}=I_{pipette}+I_{Seal}+I_{C}+I_{Access}\;$$

To simplify Equation (5), *I_Pipette_* can be cancelled out using a setting of the patch clamp. In addition, *I_Seal_* is usually small enough to be neglected when *R_Seal_* is very large. *I_C_* is nonzero only when *V_m_* is changing [23]. Throughout the microinjection, *V_m_* is controlled at a constant level. When studying the ion channel activities, the stimulus of a square voltage pulse is applied to the cell. Hence, *I_C_* will be nearly zero because *V_m_* is always constant and *V_m_* only changes at the brief instants when the voltage is stepped to a new value. In other word, $I_{C}=C_{m}\frac{dV}{dt}\;≅0$ under the stimulus of a square voltage pulse. *I_Access_* is composed of the current passing through the sodium, potassium, and other ion channels (*I_Na_*, *I_K_* and *I_Other ions_*) [25], as well as the current drop due to the injection (*I_Injection_*) in Equation (6).

(6) $$I_{Access}=I_{Na}+I_{K}+\;I_{Other\;ions}+I_{Injection}$$

By combining Equations (5) and (6), the membrane current (*I_M_*) will be directly equal to *I_Access_*, as shown in Equation (7).

(7) $$I_{m}=\;I_{Access}=I_{Na}+I_{K}+\;I_{Other\;ions}+I_{Injection}$$

**Figure 3:**
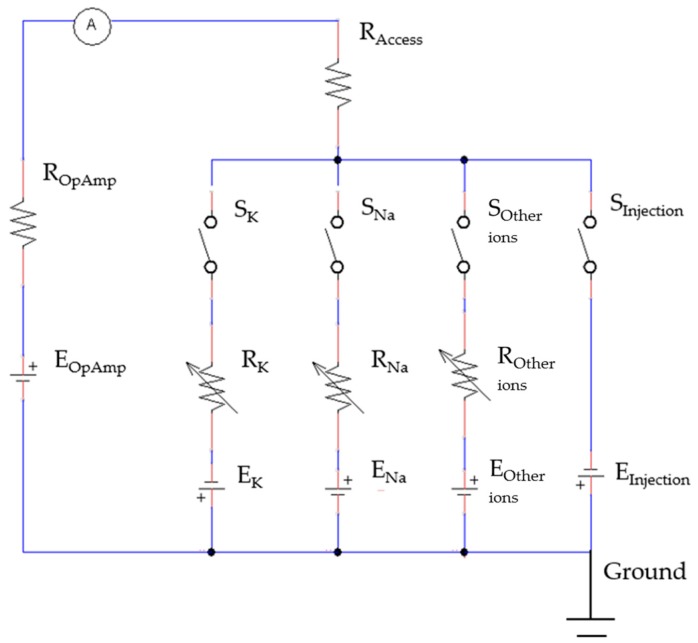
The simplified circuit of the current feedback model for the cell cytoplasmic microinjection and cell viability verification.

Therefore, the electric equivalent circuit of the cell cytoplasmic microinjection can be simplified as shown in Figure 3.

### 2.1. The Equivalent Model for the Cell Cytoplasmic Microinjection

The microinjection was performed on a single living cell when it was at the resting state. *S_K_*, *S_Na_*, and *S_Other ions_* are anticipated to be open because *R_Na_*, *R_K_*, and *R_Other ions_* are expected to be very large since nearly none of the ion channels will be activated without the voltage stimulus. Hence, *I_Na_*, *I_K_*, and *I_Other ions_* will become zero. Subsequently, *I_M_* will only be equal to the current drop, as *R_Access_* is connected to *E_Injection_*, induced by the concentration difference of the ions in the cytoplasm of the living cell due to the microinjection of the intracellular solution, as shown in Equation (8).

(8) $$I_{m}=I_{Injection}=\frac{E_{Injection}}{R_{Access}}$$

### 2.2. The Electric Model for Cell Viability Verification

After the injection to a living cell, its vitality and viability are of great concern and can be verified by the study of ion channel activities. When the cells are alive after the microinjection, the activities of the ion channels can be activated by applying the voltage stimulus. Under the voltage stimulus, the cell membrane current *I_M_* of the normal cell can be calculated by Equation (9). In this situation, *R_K_*, *R_Na_*, and *R_Other ions_* are very low, so those ions can easily pass through the cell membrane when the Na^+^, K^+^, and other ion channels are activated. Moreover, *I_Other ions_* can be neglected when compared with *I_Na_* and *I_K_* because the activities of the Na^+^ and K^+^ ion channels are dominant. Therefore, the *I_M_* of a normal cell is equal to the sum of *I_Na_* and *I_K_*, as shown in Equation (10).

(9) $$I_{m}=I_{Na}+I_{K}+\;I_{Other\;ions}$$

where $I_{Other\;ions}≅0$.

(10) $$I_{m}=I_{Na}+I_{K}$$

where the current response of *I_K_* and *I_Na_* depends on *R_K_* and *R_Na_*, respectively, as shown in Equations (11) and (12).

(11) $$I_{K}=\frac{V_{m}-E_{K}}{R_{K}}$$

(12) $$I_{Na}=\frac{V_{m}-E_{Na}}{R_{Na}}$$

*R_K_* and *R_Na_* are variables based on the number of activated ion channels for passing Na^+^ and K^+^ ions across the membrane.

## 3. The Experimental Setup of Cell Cytoplasmic Microinjection

**Figure 4:**
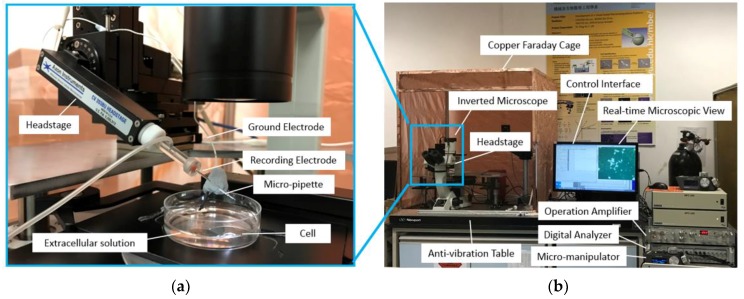
The experimental setup of the current feedback injection: (**a**) the close-up view; (**b**) the overal view.

The experimental setup consists of several units (as shown in Figure 4), which are an extracellular solution to bath the cell in order to provide cells with an in vitro environment like in vivo, an intracellular solution to act as an electrolyte connected to a low-noise amplifier and to be injected inside the cell during the microinjection process, a low-noise amplifier (Model: Axopatch 200B from Molecular Device, Sunnyvale, CA, USA) to amplify the recording current signal from the cell, a data digitalizer (Digidata 1440A from Molecular Devices) and computer for generating the voltage stimulus waveform and data acquisition of the current signal from the cell membrane, and the inverted microscope (Model: Eclipse Ti from Nikon, Tokyo, Japan) to monitor the cell cytoplasmic microinjection. The adherent cells grown on cover slips were prepared. In each experiment, the cover slip with adherent cells was placed inside a petri dish, filled with extracellular solution. When implementing the microinjection, the force applied on the cell via the micropipette was vertical, which would not lead to the sliding of the cover slip and movement of the cell. In addition, a Faraday cage is used to reduce the background signal in the surrounding area since the patch clamp recording is very sensitive to background noise.

### 3.1. The Cell Cytoplasmic Microinjection Process

In this process, the cell cytoplasmic micro-injection process was divided into five main steps. Firstly, a micropipette was made from borosilicate glass capillaries (BF120-69-7.5 from Sutter Instrument, Novato, CA, USA). Each glass capillary was pulled by a CO_2_ laser-based micro-pipette puller (Model: P-2000 from Sutter Instrument). As a result, the glass capillaries were divided into two halves, which were the micropipettes with the tip size of 1 µm. From the literature, the common tip size ranges from 0.05 µm to 1 µm [11,17,19,22]. Secondly, SHSY-5Y cells were weekly prepared by cell passage and cell culture. Thirdly, the extracellular solution and the intracellular solution were prepared with various ions. The details of the cell culture and the solution preparation will be shown in the coming sections. Fourthly, the injection process was initiated by positioning the micro-pipette filled with intracellular solution to approach the cell surface using the micro-manipulator. The electrode placed inside the micropipette was connected to the headstage and hence to the operational amplifier. A test square voltage pulse of 20 mV for 10 ms was applied to the cell from the operational amplifier.

When the pipette was immersed into the solution, a typical positive pressure was applied using a 10 mL syringe by using a displacing plunger of about 1 mL to remove any contaminations at the tip. The resistance value was around 4–8 MΩ at the bath position. Afterwards, the pipette tip slowly approached the cell surface until there was an obvious increase of 3 to 5 MΩ in the total resistance and a significant decrease in the test pulse amplitude. When the current signal became steady, the positive pressure could be released rapidly. By applying suction through retracting air using a 5 mL syringe, the plunger was retracted at 0.1 mL/s for a second. Consequently, a very tight seal between the pipette and cell surface can be created with a GΩ formation where the total resistance of the electrical model will be around 1 GΩ. Afterwards, the voltage potential of the cell membrane was controlled at −70 mV and a stronger pressure was applied to the micropipette through retracting air using a 5 mL syringe. The plunger was retracted at 1 mL/s until a significant change of the current response was observed with the existence of capacitive current trace from the cell membrane. In the circuit, the membrane current was measured spontaneously and continuously while the voltage potential was kept at −70 mV throughout the microinjection process. Before starting the microinjection experiment, the baseline of the electric current response was always adjusted to zero. Consequently, the current due to the microinjection can be acquired without the distortion from the original ion activities.

During the microinjection, the current signal of the cell membrane was monitored. The permanent drop of current level was consistently observed right after each injection. In each injection, the injection volume was estimated to be 19 pL. The estimation was based on recording the volume of the cell cytoplasm before and after the injection using optical images. In another study, the injection volume for cells (25 µm) was measured and ranged from 2 to 22 pL [11]. Moreover, the patch clamp technique was used to compare the cell condition before and after the injection, and it can be done by applying a series of voltage square pulses from the operational amplifier.

### 3.2. The Patch Clamp Technique

The principle of the patch clamp technique is to isolate a patch of membrane electrically from the external solution and to record the current flowing into the patch. During each recording, a fire-polished glass pipette filled with a suitable electrolyte solution was pressed against the surface of a cell whilst applying light suction to create a seal whose electrical resistance is more than 1 GΩ, called the gigaseal formation [25]. The patch clamp technique can be carried out under different kinds of configurations, which can record the total current of ion channels on the cell membrane of the intact cell at the whole cell mode [26]. The patch clamp allows an investigation of the change of the cell state from resting potential to action potential and the functional properties of ion channels. The action potential is a transient, regenerative electrical impulse in which the membrane potential (*V_m_*) rapidly rises to a positive voltage value from a negative voltage value, namely the resting potential where the cell is at its resting state [27]. Under a voltage stimulus, the ion channels of the living cell normally respond to generate an electric impulse. The method is believed to be a more precise and accurate way to determine the cell condition.

### 3.3. SHSY-5Y Cells and HEK-293 Cells

In this study, a SHSY-5Y cell, which is the human derived neuroblastoma cell, and a HEK-293 cell, which is a human embryonic kidney cell, were used to study the performance of the microinjection. The SHSY-5Y cell is often used as in vitro model to study Parkinson’s disease through studying the ion channel activities. The HEK-293 cell is widely used in cell biology, including in the study of gene therapy, and is also used as one mammalian expression system in the study of voltage-gated K^+^ channels [28,29]. The cells were cultured in standard conditions (Dulbecco’s Modified Eagle’s Medium supplemented with 10% fetal bovine serum and 1% penicillin-streptomycin). The cells were weekly passaged via detachment with trypsin-EDTA, 5-min centrifugation at 1900 rpm, resuspension of the pellet in a 25 mL flask filled with 3 mL of medium, and seeding of a new flask with 100–200 μL of suspension.

### 3.4. Intracellular and Extracellular Solution

During the micro-injection, the cell lines cultured on a cover slip were bathed in extracellular solution containing 160 mM NaCl, 4.5 mM KCl, 1 mM MgCl_2_, 2 mM CaCl_2_, 5 mM of glucose, and 10 mM of HEPES with pH adjustment to pH 7.4 using NaOH. The intracellular solution was prepared using 75 mM KCl, 10 mM NaCl, 70 mM KF, 2 mM MgCl_2,_ 10 mM HEPES, and 10 mM EGTA with pH adjustment to pH 7.2–7.4 using KOH.

## 4. The Current Response of the Cell Cytoplasmic Microinjection

### 4.1. The Current Drop of the Cell Cytoplasmic Injection

**Figure 5:**
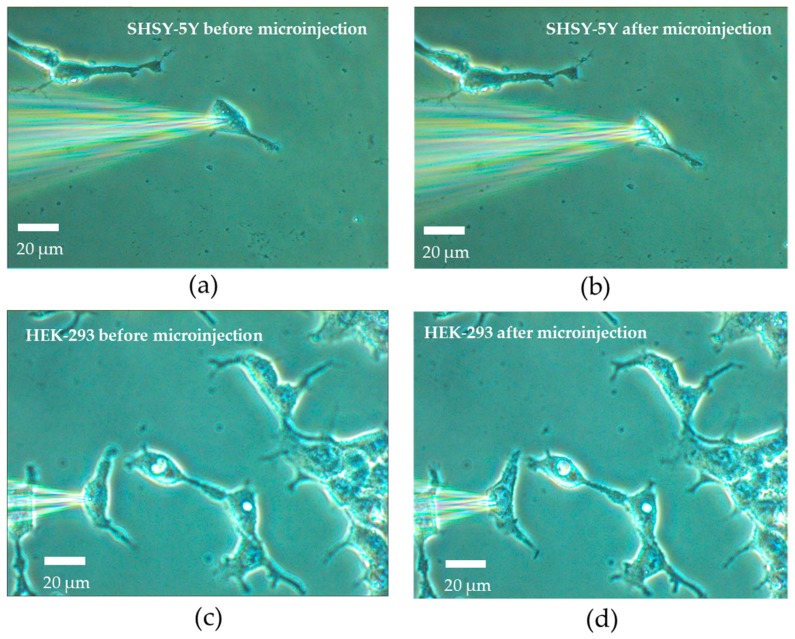
Photos of the micropipette and the cells: (**a**) SHSY-5Y cells before the microinjection; (**b**) SHSY-5Y cells after the microinjection; (**c**) HEK-293 cells before the microinjection; (**d**) HEK-293 cells after the microinjection.

The cells were placed at the stage of the inverted microscope and optical images were obtained during the injection process, as shown in Figure 5. In each injection, a single cell was selected and a tight seal was formed between the micropipette and the cell surface. Then, the intracellular solution was injected into the cell by injecting around 19 pL intracellular solution.

**Figure 6:**
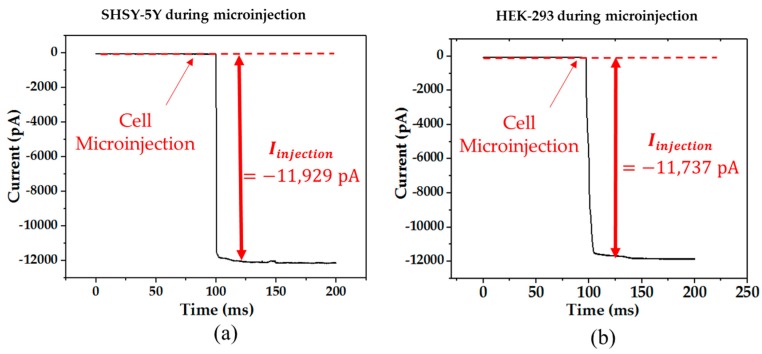
The current response during the cell cytoplasmic microinjection of: (**a**) a SHSY-5Y cell and (**b**) a HEK-293 cell.

In this work, different batches of SHSY-5Y cells and HEK-293 cells were employed in the experiment. In each batch, one single cell was selected. To elucidate the current response of the SHSY-5Y cells and the HEK-293 cells during the microinjection, current responses of the SHSY-5Y cell and the HEK-293 were recorded as shown in Figure 6. Since the estimated injected volume was 19 µL, the new concentration of [K^+^] and [Na^+^] were 150.5 µM and 15.1 µM, respectively, and the *E_injection_* was estimated at −0.3 mV, which was not sufficient to activate the ion pumps (<10 mV) after the injection. As a result, *E_Injection_* would contribute a current drop during the injection. In this experiment, the accumulated current drop is significant at −11.9 nA and −11.7 nA for the SHSY-5Y cells and HEK-293 cells, respectively.

### 4.2. The Current Response for Verifying the Cell Viability after the Cell Cytoplasmic Microinjection

**Figure 7:**
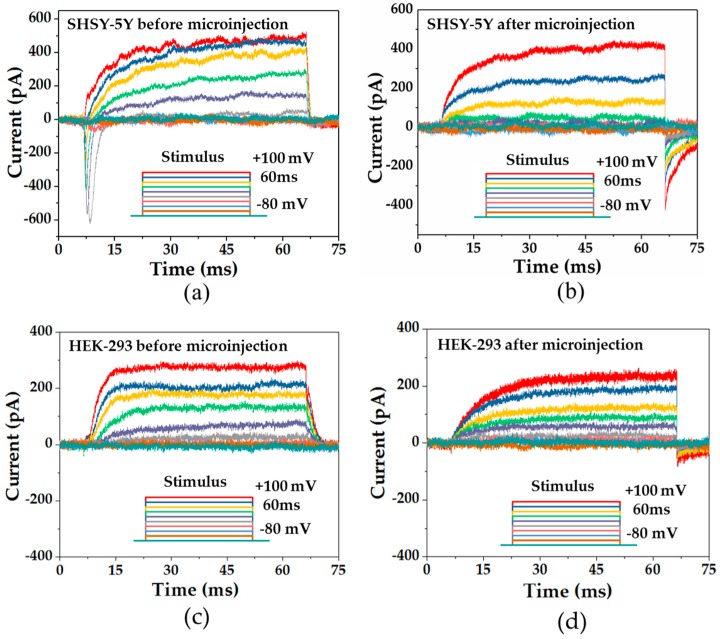
The current traces of ion channels and the voltage stimulus of a SHSY-5Y cell: (**a**) before the microinjection and (**b**) after the microinjection. The current traces of ion channels and the voltage stimulus of a HEK-293 cell: (**c**) before the microinjection and (**d**) after the microinjection.

Before and after the microinjection, electrical responses of SHSY-5Y cells were recorded as shown in Figure 7a,b. Similarly, the electrical responses of HEK-293 cells were recorded as shown in Figure 7c,d. The responses of both cells were observed under the voltage stimulus of a square pulse from −80 mV to 100 mV with the epoch of 60 ms. The current responses can be studied through the direction and the amplitude of the current trace.

Before the microinjection, the observation of the normal ion channel activities of both Na^+^ and K^+^ ion channels in SHSY-5Y cells were known with the inward current due to the hyperpolarization of Na^+^ ion channels and the outward current due to the depolarization of K^+^ channels that occurred at the same time, which was also reported by another research group [27]. Meanwhile, the normal ion channel activities of the K^+^ ion channels were recorded in HEK-293 before the microinjection.

After the microinjection, the viability can be verified by the current response, due to the ion channel activities evolved by the voltage stimulus of a square pulse from −80 mV to 100 mV with the epoch of 60 ms, applied from the low noise amplifier. The ion channel activities of K^+^ ion channel were found to be normal with the outward current due to the depolarization of K^+^ channels [27]. The current responses were compared before and after the microinjection of SHSY-5Y cells and HEK-293 cells. In SHSY-5Y cells, K^+^ ion channels can be activated in both circumstances, while Na^+^ ion channels can only be activated before the microinjection. Due to the microinjection, Na^+^ ions were redistributed across the cell membrane, while Na^+^ ions inside the cell membrane were consumed and hence Na^+^ ion channels could not be activated after the microinjection. However, the disappearance of Na^+^ ions did not lead to the death of the cell, which can be proven by the sustainable activities of the K^+^ ion channels. It is believed that we can re-activate the Na^+^ ion channels if we inject more Na^+^ into the cell. In HEK-293 cells, K^+^ ion channels can be activated in both circumstances.

### 4.3 Reliability and Repeatability of the Microinjection

**Figure 8:**
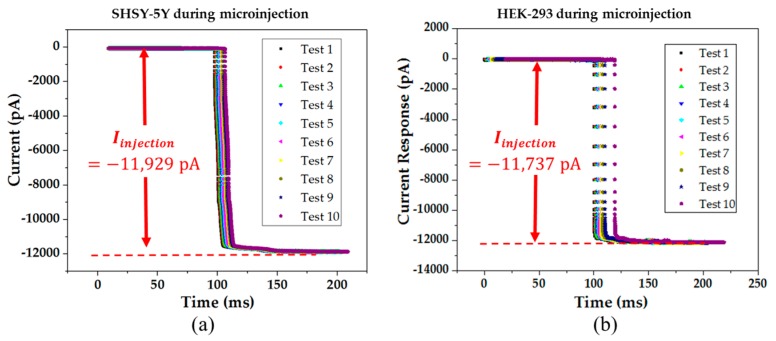
The current response of the cell during the cell cytoplasmic microinjection: (**a**) the microinjection process of the SHSY-5Y cell with 10 results; (**b**) the microinjection process of the HEK-293 cell with 10 results.

In the experiment, the reliability and repeatability are two important indicators. The reliability can be found by the consistency of the total current drop after the injection, whilst the repeatability can be demonstrated by the similar results of two different cells, as shown in Figure 8. We found that the current response of SHSY-5Y cells and HEK-293 cells are (−11.9 ± 0.2) nA and (−11.7 ± 0.4) nA, respectively.

**Figure 9:**
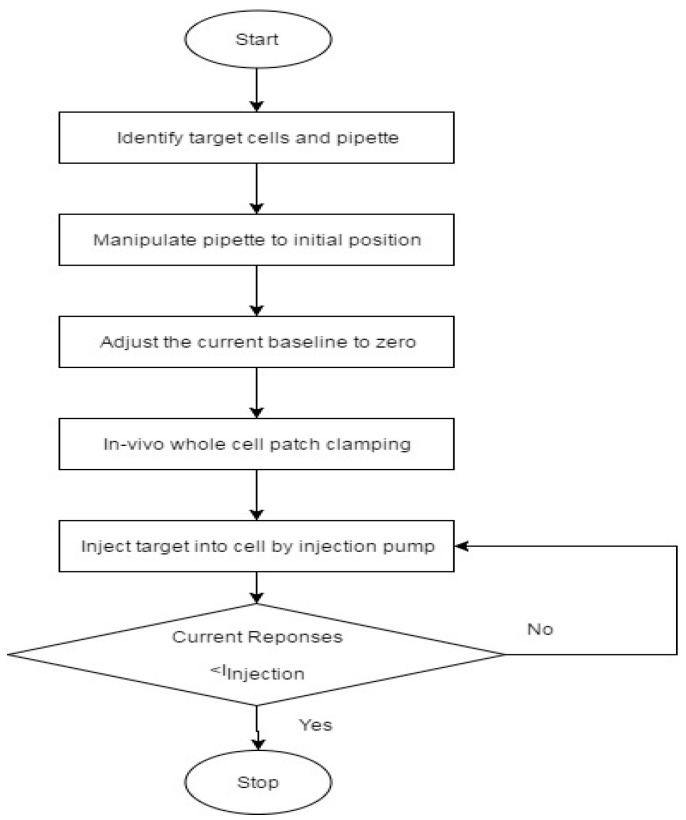
Control strategy of the microinjection system.

Based on the electrical equivalent model, a conceptual control strategy for the microinjection was considered, as shown in Figure 9. Firstly, the position of the cell and pipette were obtained via the visual-based cell and pipette segmentation process, which was developed in our group [30]. Subsequently, the pipette was manipulated to a position on the boundary of the cells, and the automated in vivo whole patch clamping process was then started, which was also developed in our group [31].

Afterwards, the current response during the microinjection was recorded, and the current value was compared with a threshold value. The threshold value is a current drop during the injection (*I_Injection_*), which we always found during the cell injection. In our experiments, *I_Injection_* was found to be at around −11 nA. A current response lower than the threshold can indicate a successful microinjection. In the near future, we will focus on realizing the control strategy in order to build an automated control system.

## 5. Conclusions

An electric model for a cytoplasmic microinjection with current feedback was designed especially for small adherent cells. To elucidate the model for realizing the current feedback, the cytoplasmic microinjection was performed on the SHSY-5Y cell and the HEK-293 cell. During the microinjection, the consistent current drop (~12 nA) was found on both cells across the cell membrane with a prompt response within several milliseconds for the SHSY-5Y cell and the HEK-293 cell, while the voltage potential of the cell membrane was maintained at −70 mV. The viability of cells after the cytoplasmic microinjection can be well proven by the sustainable activities of K^+^ ion channels. In the future, the intracellular solution could act as a transporter of various drugs or other target materials for the micro-injection of small adherent cells using this technique.
